# Pattern of play behavior in infant (age 1 to 12 months) white‐headed langurs in limestone forests, southwest China

**DOI:** 10.1002/ece3.9160

**Published:** 2022-08-04

**Authors:** Liting Yang, Tao Sun, Yingming Zhou, Chuangbin Tang, Chengming Huang, Penglai Fan, Qihai Zhou

**Affiliations:** ^1^ Key Laboratory of Ecology of Rare and Endangered Species and Environmental Protection (Guangxi Normal University), Ministry of Education Guilin Guangxi China; ^2^ Guangxi Key Laboratory of Rare and Endangered Animal Ecology College of Life Sciences, Guangxi Normal University Guilin Guangxi China; ^3^ College of Chemistry and Bioengineering, Guangxi Normal University for Nationalities Chongzuo Guangxi China; ^4^ Institute of Zoology, Chinese Academy of Sciences Beijing China

**Keywords:** age specificity, motor play, social play, *Trachypithecus leucocephalus*

## Abstract

Play behavior is a significant trait of immature nonhuman primates (hereafter primates) that plays an important role in sensory, locomotor, socio‐cognitive, and developmental processes. It has been suggested that the function of play is to practice and improve motor skills related to foraging, avoiding predators, attracting mates, raising offspring, and strengthening the skills needed for the formation and maintenance of social bonds. From September 2009 to August 2010, we investigated the play behavior of 1–12 month‐old infant white‐headed langurs (*Trachypithecus leucocephalus*) a Critically Endangered primate species endemic to China. We carried out this study in the Guangxi Chongzuo White‐headed Langur National Nature Reserve, and recorded 4421 play bouts and 1302 min of play engaged in by seven infants. We found that infants of different ages exhibited different patterns of play behavior. Specifically, nonsocial play behaviors appeared at one month of age, social play behaviors at two months, and an expanded repertoire of social and non‐social play behaviors at three months of age. The frequency and duration of nonsocial play peaked at five months of age and then decreased, while social play gradually increased with age. Nonsocial play did not differ between the sexes, whereas social play showed sex specificity, with a higher frequency and longer duration of social play in male infants compared to female infants. In addition, male and female white‐headed langur infants appeared to prefer individuals of same sex as social playmates, but no obvious choice preference for a specific individual. In conclusion, we provide the first report of play behavior in a population of wild Critically Endangered white‐headed langurs. We suggest that age‐ and sex‐specific differences in play behavior of infants form the bases for age and sex‐based differences in the social interactions of adult langurs.

## Graphical abstract

We found that infant white‐headed langurs had different play behavior patterns in different ages. Specially, the frequency and duration of non‐social play peaked at 5 months old and then decreased, while social play appeared at 2 months old and gradually increased with age. Non‐social play showed no differences between the sexes, whereas social play showed sex specificity, with a higher frequency and duration of social play in male infants than in female infants. In addition, male and female white‐headed langur infants preferred the individuals of same sex as social playmates.

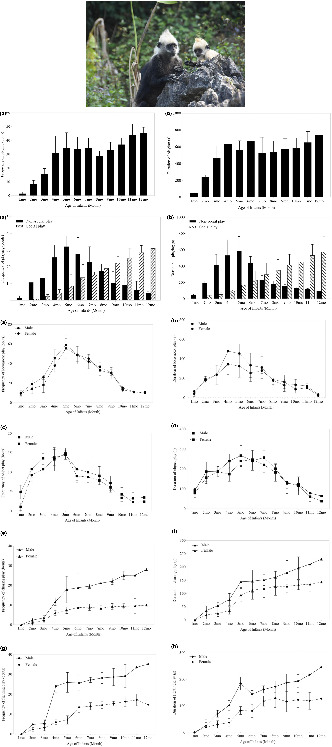

## 1 INTRODUCTION

Play behavior is repeated, seemingly nonfunctional behavior differing structurally, contextually, and developmentally from other forms of behavior, and is initiated when the individual is in a relaxed, unstimulating, or low stress setting (Burghardt, 2014). In nonhuman primates (hereafter primates), play is a significant and crucial behavior for proper physical and social development (Goodall, 1968; Perret, 2020). It has been reported that play behaviors include nonsocial play and social play (Burghardt, 2014; Maestripieri & Ross, 2004). Nonsocial play refers to spontaneous solitary play activities were exhibited by immature animals that occur without social interaction. Nonsocial play may serve to improve survival skills and the acquisition of new behavioral patterns (Berghänel et al., 2015). In contrast, social play is defined as playful interactions involving two or more individuals that may be beneficial to establishing and maintaining social relationships (Gennuso et al., 2018). Studies have shown that play behaviors play an important role in sensory, locomotor, socio‐cognitive, and developmental processes (Fagen, 1981; Maestripieri & Ross, 2004). In addition, play can act as a buffer against social pressures, and can facilitate the establishment and enforcement of social relationships or alliances with conspecifics that increase access to food resources and future breeding opportunities (Jiang, 2010; Karimullah et al., 2014; Li, Guo, et al., 2011; Li, Ren, et al., 2013; Lutz & Judge, 2017; Palagi, 2018; Schneider et al., 2010). For example, infants practice and enhance physical flexibility, eye–hand coordination, and fighting skills through play behaviors (Maestripieri & Ross, 2004). Moreover, play behavior may promote the development of the cortico‐cerebellar system of primates (Kerney et al., 2017), and contribute to the emergence of the unique skills needed for survivorship (Maestripieri & Ross, 2004). For example, juvenile Burmese long‐tailed macaques (*Macaca fascicularis aurea*) are reported to increase their foraging skills through solitary object play (Haslam et al., 2016). Similarly, gorillas (*Gorilla gorilla*) who play more during their adolescent period display greater physical strength and competitiveness in adulthood (Lutz & Judge, 2017; Palagi, 2018). Thus, changes in the patterns and types of play behavior engaged in by infant, juvenile, and adolescent primates, have a significant effect on adult behavior (Maestripieri & Ross, 2004).

Over the course of infant development, play behavior is influenced by ages and the actions of both the infant and its mother (Byers & Walker, 1995; Palagi et al., 2006; Shimada, 2006; Zhao et al., 2008). Generally, play behavior begins in infancy, reaches a peak during the juvenile period, and then declines steadily during adolescence (Fagen, 1981). For example, golden snub‐nosed monkeys (*Rhinopithecus roxellana*) mainly engage in nonsocial play behaviors such as tree climbing and grasping at 0–2 months of age, and show significantly increased social play after four months of age (Li, Guo, et al., 2011). Similarly, black‐and‐white snub‐nosed monkeys (*Rhinopithecus bieti*) first engaged social play around two months of age. These behaviors became more frequent and replaced gradually nonsocial play in the third month of life (Li, Ren, et al., 2013).

In addition, play behavior in many primates is reported to vary by sex (Barrett et al., 1992; Fagen, 1993; Maestripieri & Ross, 2004). In response to differences in sexual dimorphism, males and females exhibit sex‐specific differences in patterns of growth and development, age at sexual maturity, and have different social roles within their group (Fagen, 1993; Maestripieri & Ross, 2004). Thus, sex‐specific patterns of a play behavior may facilitate the acquisition of knowledge and abilities that enhance survival and reproduction in adults (Maestripieri & Ross, 2004). Specially, males and females face different social stresses that are associated with their adult roles (Nikolei & Borries, 1997). Males face the pressure of male–male competition and controlling access to mates, whereas females devote considerable time and energy to producing and caring for offspring (Jin et al., 2009). In female philopatric species, females also spend considerable time engaging in social interactions that serve to maintain long‐term relationships with related females (Glick et al., 1986). Therefore, we might expect males to spend more time engaging in behaviors designed to improve motor coordination and strength, whereas females are expected to spend more time engaging in behaviors designed to maintain social relationships and skills that enhance their ability to raise offspring (Zhao et al., 2009). For example, male infant and juvenile gorillas show a higher frequency of social play than females (Maestripieri & Ross, 2004). Similar results are also reported in male infant Francois' langurs (*Trachypithecus francoisi*) (Li, Jiang, et al., 2013) and tufted capuchin monkeys (*Sapajus apella*) (Paukner & Suomi, 2008). In contrast, there were no significant differences in the frequency of social play between juvenile male and female Tibetan macaques (*Macaca thibetana*) (Wang et al., 2021), and female infant red colobus monkeys (*Colobus badius*) produced a higher frequency of social play than male infants (Starin, 1990).

There also is evidence that age and sex differences in the choice of play partners varies across species (Bekoff, 2001; Nicola & David, 1995; Schneider et al., 2010; Shimada & Sueur, 2018). For example, infant male Francois' langurs show a preference to play with male peers (Li, Jiang, et al., 2013). Similar findings have been reported in captive Assamese macaques (*Macaca assamensis*) (Jiang et al., 2010) and Japanese macaques (*Macaca fuscata*) (Glick et al., 1986).

**FIGURE 1:**
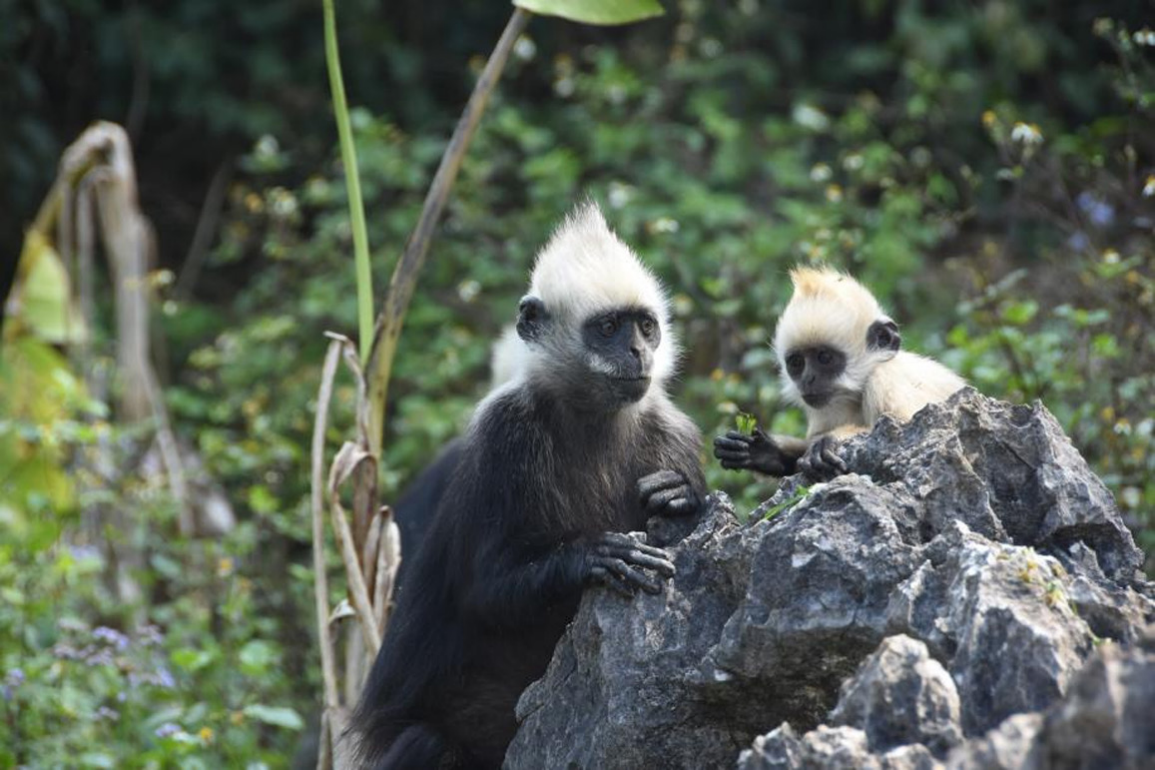
The infant white‐headed langur (*Trachypithecus leucocephalus*) is feeding beside its mother, photo by Penglai Fan.

The white‐headed langur (*Trachypithecus leucocephalus*) is a Critically Endangered primate species endemic to China (Figure 1.). Currently, they are distributed exclusively across a karst mountain area of approximately 200 km^2^ between the Zuojiang and Mingjiang Rivers in Guangxi Province, China. The population totals approximately 1200 individuals (Huang et al., 2021). They live in a typical polygynous and matrilineal social system composed of one adult male, multiple adult females and offspring (Liu et al., 2013). Adult females form the stable core membership of a group, whereas males leave their natal group around three years old and transfer into a new group. Adult males compete fiercely for reproductive opportunities (Jiang et al., 2010; Jin et al., 2009). Female gives birth for the first time at five years old, and the inter‐birth interval is 23 months (Jin et al., 2009; Pan, 2021). Infants are born throughout the year but births peak from November to March (Zhao et al., 2011). Infants begin to chew on dead plant material (dry leaves, bark, and twigs) in addition to fresh leaves at about one month old, and are fully weaned at 19–21 months of age (Zhao et al., 2008). Several studies have identified sex differences in foraging, grooming, and infant care in white‐headed langurs. For example, adult males spend more time foraging and adult females groom more frequently. Adult males are not directly involved in caring for infants (Huang & Li, 2005; Lu et al., 2016; Zhang et al., 2021; Zhao et al., 2011). Due to difficulties in observing white‐headed langurs in the wild, little is known concerning patterns of play in infants.

In this study, we examined patterns of play behavior in wild infant white‐headed langurs (aged 1–12 months). Given age and sex‐based differences in langur physical development and sexual dimorphism, we investigated the effects of infant age and sex on play behaviors. In addition, due to the fact that males migrate into new social group where they compete for access to fertile females, whereas females remain in their natal group maintain strong social relationships with closely related females (Jin et al., 2009; Zhao et al., 2011), infant males are expected to engage in play behaviors such as chasing and fighting more frequently than females, and male and female infants are expected to preferentially play with infants of the same sex.

## 2 METHODS

### 2.1 Study site and subjects

The study site is located in the White‐headed Langur National Nature Reserve (22°27'N, 107°52'E). This study area is characterized by seasonal limestone rainforest, and is a highly fragmented (Huang & Li, 2005). The forests are dominated by tree families such as Euphorbiaceae, Sterculiaceae, Opiliaceae, Meliaceae, Moraceae, Caprifoliaceae, Rhamnaceae, Annonaceae, Urticaceae, Rutaceae, Lauraceae, and Sapindaceae (Zhang et al., 2021). This area has a tropical monsoon climate of the Northern Hemisphere. The dry season ranges from October to March and rainy season is from April to September (Zhang et al., 2021). The average annual temperature is 21.6°C, the maximum monthly mean temperature is 33.3°C in July, and the minimum monthly mean temperature is 10.0°C in January. The mean annual precipitation is approximately 1200 mm, with a maximum value of 234 mm in August and a minimum value of 25 mm in December (Huang et al., 2006; Zhang et al., 2021). Seven newborn infants (three males and four females) from a habituated group of wild white‐headed langurs were the focus of this study. The study group consisted of eight adult individuals (one male and seven females) and seven infants (three males and four females). The group composition of our study group is shown in Table A1 of the Appendix. All infants were observed beginning on their first day of birth. Two infants were observed for 12 months, two infants were observed for 11 months, two infants were observed for 10 months, and one infant was observed for a period of six months (Table A2 of the Appendix). Each infant was individually identified based on their coat color, body size, and the characteristics (facial features, coat color, perineal marking) of their mothers on which the infants rely.

### 2.2 Classification and definition of play behaviors

Nonsocial and social play behaviors were defined based on a study by Fagen (1981). Nonsocial play behaviors include solitary locomotor play and object play (Fagen, 1981). Locomotor play is defined as physical activities such as climbing and jumping when infants were alone. Object play refers to the repeated manipulation of environmental objects including branches, leaves, and stones as well as their mothers' fur.

Social play primarily included infants chasing and fighting with conspecifics (Fagen, 1981; Maestripieri & Ross, 2004). During chasing play, two or more individuals followed each other for a short distance. Typically, one individual would run and then stop, look back at its playmate, and the latter would immediately run towards the former. Fighting play occurred when two or more individuals engaged in of mock wresting, biting, and mildly aggressive physical contact with another playmate. During bouts of fighting play, both players stood on their hind limbs, and faced each other with their forelimbs reaching out quickly in an attempt to grasp some part of the other's body, an individual attempted to grasp and mock bite the shoulder or head of the other individual.

### 2.3 Data collection

Play behaviors of infant white‐headed langurs were observed from September 2009 to August 2010. We used a 10‐min focal animal sampling method to record play behaviors. We scored a new bout of play when individuals changed to a new form of play, a bout ended players separated for a period of at least 30 s and then resumed play, or when the identity of play partners changed. When more than two individuals participated in the same play bout, this was scored as two or more play bouts (Barrett et al., 1992; Jiang et al., 2010). For example, when three individuals play together, it would considered two play bouts for each individual. We numbered each infant and selected the nearest and most easily observed infant for data collection. When we finished a 10‐min sampling period, we selected the nearest infant who was not the previous focal animal and recorded its play behavior. We used this rule to collect play behavior for all infants in as equal frequency as possible. If the infants were hidden in caves or vegetation during periods of high temperature, heavy rain, or the presence of predators, the observation was terminated.

### 2.4 Data analysis

Because the frequency and duration of observation periods varied across individuals, we calculated the monthly play frequency and duration for each infant. We then divided that value by the total monthly observation time for each individuals, and calculated the relative play frequency and duration for each individual. The mean relative play frequency and duration for infants of the same age was calculated as the total play frequency and duration for individuals of the same age divided by the number of individuals of that same age class.

We first used the Shapiro–Wilks test to assess whether the data was normal distribution. According to the data did not conform to a normal distribution (frequency: *p* = .012; duration: *p* < .001), we log‐transformed the non‐normal variables prior to data analysis. Second, we used Linear Mixed Models (LMMs) to test whether there are differences in the relative frequency (and duration) of total play behavior (Models 1 & 2), nonsocial play (Models 3 & 4) and social play (Models 5 & 6) among different months of age. For LMMs, we set up the relative frequency (and duration) as dependent variable, age of individuals as fixed effect, individual identity as random effect, and individual identity as random intercept in the model. Third, we also used LMMs (Models 7 to 14) to test the differences in the relative frequency (and duration) of four kinds of play behaviors (locomotor play, object play, chasing play, and fighting play) across different sexes. In these models, we used the relative frequency (and duration) as dependent variables, the sex and age as fixed effects, individual identity as random effect, and the individual identity as random intercept. We also used the values of AIC and BIC as the basis for selection, when choosing the optimal models. Finally, we used Chi‐square test to assess the differences in the sexes of selected playmates between male and female individuals. Then, we used the same method to test whether infants prefer to play with specific individuals. All statistical operations were performed in R version 3.6.1 (R Core Team, 2021), using the packages “lme4” and “lmerTest” (version 1.1–19) for running LMMs and the packages “stats” (version 4.1.2) for running Chi‐square test (Bates et al., 2012; Kuznetsova et al., 2013), and statistical significance was set at *p* < .05 with two‐tailed.

## 3 RESULTS

### 3.1 Changes in play behaviors across different ages

**FIGURE 2:**
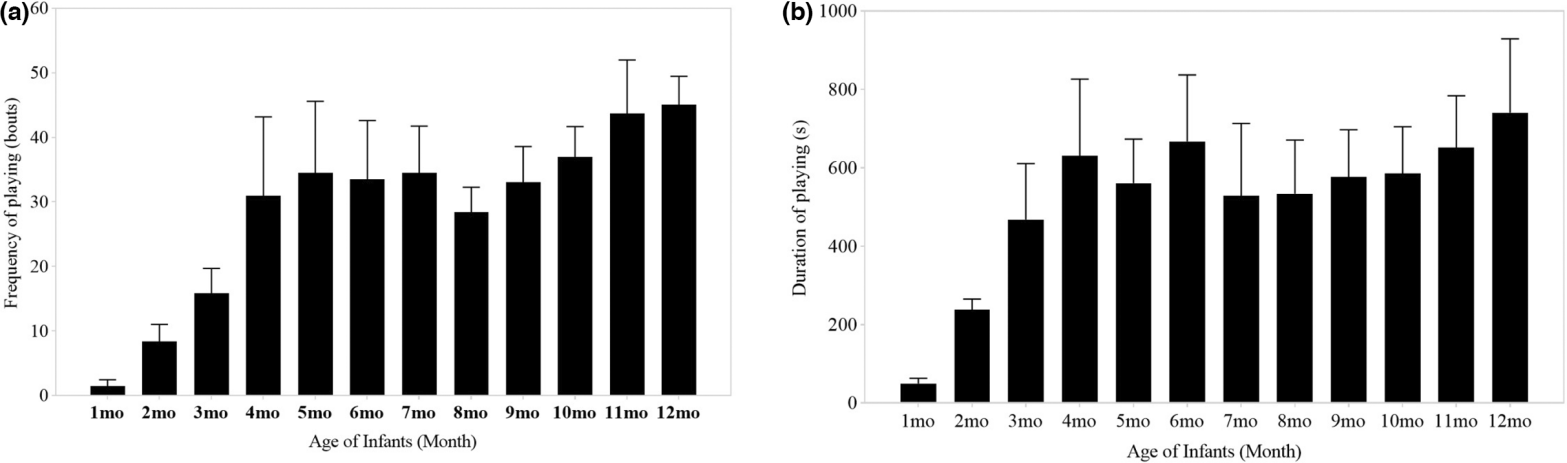
Variation in frequency (a) and duration (b) of play behaviors of infant white‐headed langurs among different months of age. Seven infants were studied for 1–6 months, six for 7–10 months, four for 11 months, and two for 12 months. Data was shown by mean ± SD.

During this study, we observed infant langurs for a total of 4088 min, of 33.6% was devoted to play. The observation time for each infant was shown in Table A3. We recorded 4421 play bouts (mean ± SD = 632 ± 178, range: 334–1001) and 1302 minutes of play time (mean = 186 ± 72, range: 88–325). Nonsocial play accounted for 56.7% of play bouts and social play accounted for 43.3%. In total, 44.5% of these bouts involved locomotor play, 12.6% object play, 17.9% chasing play, and 25.4% fighting play. In terms of time participated in play behavior, nonsocial accounted for 55.1% and social play accounted for 44.9%. Based on play time, 34.5% involved locomotor play, 20.5% object play, 18.8% chasing play, and 26.1% fighting play. We found significant differences in the frequency (and duration) of play behaviors among 1–12 months old infant white‐headed langurs (frequency: 2.10 ± 0.23, estimate ± SE, *p* < .001; duration: 2.18 ± 0.36, *p* < .001; Figure 2a,b; Table 1: Model 1 & 2). Overall, both the frequency and duration of play behavior increased with age.

**TABLE 1:** Results of LMMs used to test for the differences in the relative frequency and duration of play behaviors among different ages or sexes (All results of the 14 models are shown in Table A4).

Model	Play types	Variables	Factors	Estimate	*SE*	AIC	95% CI	*df*	*t*	*p* value
1	Total play behavior	Frequency	Age	2.10	0.23	373.35	[0.38, 0.77]	67	8.98	<.001
2	Total play behavior	Duration	Age	2.18	0.36	373.35	[0.56, 0.89]	71	5.98	<.001
3	Non‐social play	Frequency	Age	1.52	0.27	331.55	[0.35, 0.75]	67	5.59	<.001
4	Non‐social play	Duration	Age	1.75	0.38	380.89	[0.26, 0.69]	71	4.56	<.001
5	Social play	Frequency	Age	3.07	0.17	265.70	[0.80, 1.01]	65	18.44	<.001
6	Social play	Duration	Age	3.62	0.28	335.26	[0.71, 0.97]	71	13.01	<.001
7	Locomotor play	Frequency	Sex	0.79	0.68	362.62	[−0.33, 0.09]	72	−1.16	.250
8	Object play	Frequency	Sex	0.53	0.89	379.47	[−0.31, 0.17]	72	−0.60	.567
9	Chasing play	Frequency	Sex	1.81	0.39	391.01	[−0.36, 0.05]	72	4.65	<.001
10	Fighting play	Frequency	Sex	2.20	0.77	380.08	[−0.50, −0.08]	72	−2.87	.005
11	Locomotor play	Duration	Sex	0.91	0.96	412.32	[−0.33, 0.12]	72	−0.95	.348
12	Object play	Duration	Sex	0.02	1.26	447.42	[−0.24, 0.24]	67	0.02	.987
13	Chasing play	Duration	Sex	2.05	0.55	441.54	[−0.37, 0.07]	72	3.71	<.001
14	Fighting play	Duration	Sex	1.34	0.55	441.18	[−0.43, 0.03]	72	2.43	.018

*Note*: SE means standard error; AIC is akaike information criterion and a measure of the goodness of fit in statistical models; CI means confidence interval.

**FIGURE 3:**
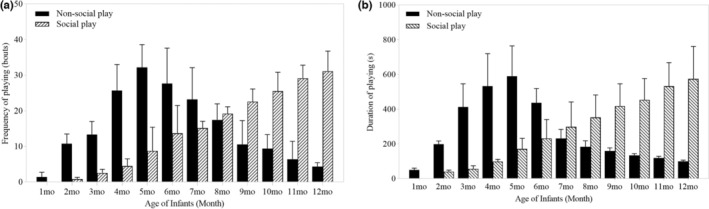
Variation in frequency (a) and duration (b) of non‐social play and social play of infant white‐headed langurs at different age in the first year. Seven infants were studied for 1–6 months, six for 7–10 months, four for 11 months, and two for 12 months. Data was shown by mean ± SD.

Furthermore, there were significant differences in the frequency and duration of nonsocial and social play behaviors across different age classes (frequency of nonsocial play: 1.52 ± 0.27, *p* < .001, Table 1: Model 3; duration of non‐social play: 1.75 ± 0.38, *p* < .001, Table 1: Model 4; frequency of social play: 3.07 ± 0.17, *p* < .001, Table 1: Model 5; duration of social play: 3.62 ± 0.28, *p* < .001, Table 1: Model 6; Figure 3a,b). The frequency and duration of non‐social play peaked at five months of age (frequency: 32 ± 6 times; duration: 591 ± 174 s) and then decreased within increasing age. Social play behaviors appeared at two months of age and then gradually increased over the 12 month period. Infant white‐headed langurs increased their social play but decreased nonsocial play after they were five months old.

### 3.2 Sex differences in the play behaviors

**FIGURE 4:**
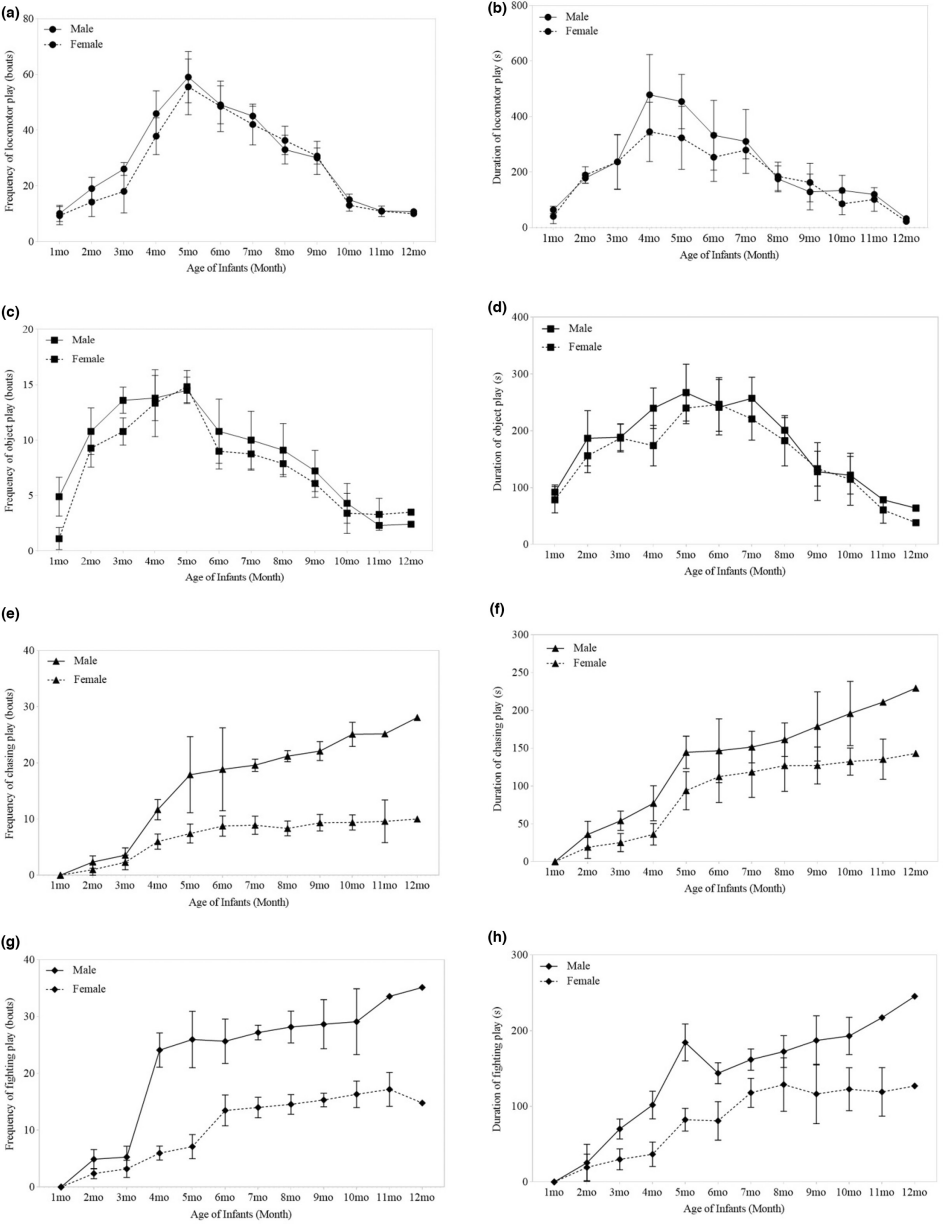
Variation in frequency and duration for four kinds of play behaviors between infant male and female white‐headed langurs at 1–12 months of age (a) Variation in the frequency of locomotor play; (b) Variation in the duration for locomotor play; (c) Variation in the frequency of object play; (d) Variation in the duration of object play; (e) Variation in the frequency for chasing play; (f) Variation in the duration of chasing play; (g) Variation in the frequency of fighting play; (h) Variation in the duration of fighting play. Three males and four females were studied for 1–6 months, two males and four females for 7–10 months, one male and three females for 11 months, and a male and a female for 12 months. Data was shown by Mean ± SD.

At 1–12 months of age, we found that no difference in the frequency (locomotor play: 0.79 ± 0.68, *p* = .250, Table 1: Model 7; object play: 0.53 ± 0.89, *p* = .567, Table 1: Model 8) and duration (locomotor play: 0.91 ± 0.96, *p* = .348, Table 1: Model 11; Object play: 0.02 ± 1.26, *p* = .987, Table 1: Model 12) of locomotor and object play between infant males and females (showed in Figure 4a–d). However, we observed sex‐specific differences in both the frequency (chasing play: 1.81 ± 0.39, *p* < .001, Table 1: Model 9; fighting play: 2.20 ± 0.77, *p* = .005, Table 1: Model 10) and duration (chasing play: 2.05 ± 0.55, *p* < .001, Table 1: Model 13; fighting play: 1.34 ± 0.55, *p* = .018, Table 1: Model 14) of chasing and fighting play (shown in Figure 4e– h). The frequency and duration of chasing and fighting play increased with age for both sexes. After three months of age, male infants showed a higher frequency and duration of chasing and fighting play than females.

### 3.3 Selection of social playmates

Overall, we observed that infant white‐headed langurs aged 1–12 months displayed sex preference in their choice of social playmates (Table 2). Specifically, male infants preferred other male infants as social playmates (Chi‐square test: *χ* ^2^ = 54.85, *df* = 11, *p* < .001) and female infants tended to select other female infants as social playmates (Chi‐square test: *χ* ^2^ = 46.12, *df* = 11, *p* < .001). However, there was no difference in choosing specific individuals as playing partners (*χ* ^2^ = 169.17, *df* = 168, *p* = .668).

**TABLE 2:** Matrices of frequencies of play among infants

I/R	XC ♂	DM ♀	XB ♀	TQ ♀	HM ♀	DD ♂	XY ♂
XC ♂	/	25	25	9	15	56	11
DM ♀	134	/	29	14	26	16	4
XB ♀	68	33	/	12	27	20	3
TQ ♀	16	24	30	/	45	36	7
HM ♀	33	28	30	33	/	24	3
DD ♂	82	30	18	21	25	/	17
XY ♂	44	17	10	25	15	36	/

*Note*: ♀‐female, ♂‐male, I‐Initiator, R‐Receiver.

## 4 DISCUSSION

Here, we provide the first report of play behavior in wild infant white‐headed langurs (age one month to 12 months). We found evidence of age and sex based differences in patterns of play. Nonsocial play began at a month of age, social play at two months, and the full repertoire of social and nonsocial play behaviors were present at three months of age. The frequency and duration of non‐social play peaked at age five months and then decreased, while social play first appeared at two months of age and gradually increased with age of up to 12 months. Patterns of nonsocial play showed no differences between the sexes, whereas we identified both a higher frequency and longer duration of social play in male infants than in female infants. In addition, both male and female white‐headed langur infants tended to prefer individuals of same sex as playmates.

Maternal rejection may be a contributing factor promoting behavioral development and infant independence in young primates (Nowell & Fletcher, 2007). For white‐headed langurs, maternal rejection is reported to occur when infants reach one month of age (Li & Rogers, 2004; Zhao et al., 2008). This suggests that mothers may encourage their infants to play and forage independently early in development (Nicolson, 1987). This is consistent with our observations of nonsocial play at one month of age and social play beginning at two months of age. As mothers allow their infants greater independence (Li et al., 2005), this provides infants with the opportunity to interact with a broader set of social partners, learn and practice survival skills, and establish social relationships (Li & Rogers, 2004; Worch, 2012; Zhao et al., 2008). In the case of wild infant golden snub‐nosed monkeys, six months old infants were rejected by their mother and began to forage independently. This period of maternal rejection coincided with the peak period that infants appeared social play (Li, Guo, et al., 2011).

Our study showed that in the case of white‐headed langurs, non‐social play accounted for a significant proportion of play time for infants aged 1–5 months, but decreased gradually after five months old. During this period, infants increased their time spent in locomotor play and object play. Survival skills related to reducing predation risk and increasing foraging efficiency may be obtained during nonsocial play (Kerney et al., 2017). At an early age, infant white‐headed langurs spend a large amount of time climbing, jumping, and grasping branches. This may allow them to master skills required to exploit steep cliffs without falling, locate and acquire resources, and to adapt to a karst mountain habitat (Li, Zhou, et al., 2011; Zhou et al., 2011). Previous studies have shown that many infant primates spend a large amount of time in non‐social play. For example, infant Francois's langurs spent 38.2% of time in locomotor play and 28.4% in object play (Li, Jiang, et al., 2013), while locomotor play accounted for 64.9% of the play time in infant Assamese macaques (Jiang et al., 2010).

Social play in infant white‐headed langurs first occurred at two months of age and then increased gradually to age 12 months. Social play may help infants build social relationships and practice communication skills needed to form alliances, engage in reconciliation, and de‐escalate aggression. Infant Tibetan macaques were found to similarly increase time spent in social play, which peaked at 12 months of age (Starin, 1990). Overall time engaged in play in infant white‐headed langurs remained relatively stable after five months of age, and was characterized by increasing social play and decreasing nonsocial play. This result suggests that after five months of age, infant white‐headed langurs engage in building stable social relationships that are vital for group‐living. In addition, this may indicate a trade‐off faced by infant white‐headed langurs. This trade‐off results from the need to balance the energy costs of play behavior with the energy gains derived from nursing and autonomous foraging (Li, Jiang, et al., 2013; Strier, 2018).

In most species, male and female primates serve different social functions, prompting infants to practice specific skills that will be needed in their social roles as adults (Holekamp, 1984; Mancini & Palagi, 2009; Nikolei & Borries, 1997). As a result, patterns of social play may be expected to differ between male and female infants (Nikolei & Borries, 1997). Typically, chasing and fighting play may help infants to improve anti‐predation and fighting skills and allow them to establish social relationships needed to respond to future challenges, such as obtaining reproductive opportunities (Holekamp, 1984; Mancini & Palagi, 2009). In our study, infant white‐headed langurs were found to exhibit sex‐specific patterns of social play. The frequency and duration of social play were higher in male infants than female infants. Based on the polygynous and matrilineal social system of white‐headed langurs, adult males may be expected to engage in intrasexual competition for protecting territory and mates. In contrast, female white‐headed langurs are responsible for infant care and rarely involved in territorial defense (Jin et al., 2009). New born white‐headed langurs are transported by their mother and exhibit little movement during the first week of life (Jin, 2008; Zhou et al., 2006). For white‐headed langurs, adult males rely on their ability to defend against conspecific invaders and protect group members from predators. Such skills may be first acquired during fighting play as infants. This may help to explain why infant males devoted more time to social play than females. The social system of Francois' langurs is similar to that of white‐headed langurs, and in Francois' langurs infant males had a higher frequency of social play than females (Li, Jiang, et al., 2013; Zhou et al., 2006). Similar results were observed in chimpanzees (*Pan troglodytes*) and Tibetan macaques (Palagi et al., 2004; Starin, 1990). In this regard, sex‐specific patterns of social play in white‐headed langurs may facilitate the establishment of social relationships among males and the successful integration of males into new groups (French, 1981; Zhou et al., 2007).

Playmates selection and preference in infancy may affect social relationships during adulthood (French, 1981; Imakawa, 1990; Mancini & Palagi, 2009; Perret, 2020; Zhou et al., 2011). We found that both male and female infant white‐headed langurs preferred to play with the same sex playmates. Male white‐headed langur infants may have preferred to play with male infants due to their similar body size and strength. Moreover, male infants displayed a higher frequency of chasing and fighting play which could enhance their fighting skills when facing male–male competition as adults (Pan, 2021). Male–male competition for access to fertile females has a significant impact on male reproductive success (Byrne & Suomi, 1998; Owens, 1975; Wang et al., 2020; Xiang et al., 2014). However, species in which males spend part of their life in an all‐male group or in which males are philopatric, patterns of social play during infancy is likely to play a role in their ability to form affiliatively and cooperative same‐sex social bonds as adults (Pan, 2021). White‐headed langurs live in a typical polygynous and matrilineal social system, with adult females as the stable core of the group and males dispersing into new groups (Tao et al., 2010; Zhao et al., 2011). Compared with the migrating males, resident females have close genetic relationship and maintain a social structure based on kinship (Tao et al., 2010). This may be why female infant white‐headed langurs tend to play more commonly with other infant female, many of which are likely their close relative. In many primates (e.g., *Pan troglodytes*, *Gorilla gorilla*, *Macaca assamensis*, *Rhinopithecus roxellana*), infant males prefer to play with males while infant females prefer to play with females (Li, Guo, et al., 2011; Maestripieri & Ross, 2004; Palagi et al., 2004; Starin, 1990). However, captive infant female Francois' langurs were found to select male partners, possibly because the captive environment limits their choice of playmates (Li, Jiang, et al., 2013). In addition, many studies have shown that infants prefer to play with the same‐age individuals (Jiang et al., 2010). In this regard, the timing of infants' birth may affect an infant's choice of playmates. In the present study, infant male XC and infant female DM were born at the same time. This may be the reason why XC and DM played with each other most.

In conclusion, we present the first study of play behavior in wild infant white‐headed langurs. We found that differ play behaviors in white‐headed langurs developed at different rates during their first year of life. Solitary play emerged by one month of age and social play by two months of age. Nonsocial play did not differ between the sexes, whereas social play showed sex specificity, with a higher frequency and a greater duration in male infants than in female infants. Both male and female white‐headed langur infants displayed a sex‐specific pattern of playmate choice. Our results suggest that patterns of play behavior early in development are critical for developing appropriate social skills in adulthood.

## AUTHOR CONTRIBUTIONS

**Li Ting Yang:** Writing – original draft (equal); writing – review and editing (equal). **Tao Sun:** Methodology (equal); writing – original draft (equal). **Yingming Zhou:** Data curation (equal). **Chuangbin Tang:** Data curation (equal); investigation (supporting). **Chengming Huang:** Conceptualization (equal); methodology (equal); supervision (equal); visualization (equal). **Penglai Fan:** Conceptualization (equal); formal analysis (equal); writing – original draft (equal); writing – review and editing (equal). **Qihai Zhou:** Conceptualization (equal); supervision (equal); visualization (equal).

## CONFLICT OF INTEREST

The authors declare that they have no conflict of interest.

## ANIMAL CARE AND ETHICAL EXAMINATION CERTIFICATE

The animal experimental design scheme was approved by the Laboratory Animal Care and Animal Ethics Committee of Guangxi Normal University, the ethical review acceptance number (202109–001).

## Data Availability

The data that support the findings of this study are openly available in Dyrad, https://doi.org/10.5061/dryad.tmpg4f50t.

## APPENDIX A

**TABLE A1:** Group composition of adult white‐head langurs in our study group.

No.	1	2	3	4	5	6	7	8
Name	DB (Dao Ba)	HT (Hei Tou)	XB (Xiao Bai)	SH (San Hua)	JM (Jian Mao)	DY (Du Yan)	HB (Hua Bi)	JG (Ji Guan)
Sex	Male	Female	Female	Female	Female	Female	Female	Female

**TABLE A2:** Basic information of infant white‐head langurs in our study group.

No.	Name	Birthday	Sex	Mother	Age range during observation
1	XC (Xiao Chou)	09‐05‐2009	Male	HT (Hei Tou)	1–12 months old
2	DM (Duan Mian)	09‐15‐2009	Female	XB (Xiao Bai)	1–12 months old
3	XB (Xiao Bao)	10‐10‐2009	Female	SH (San Hua)	1–11 months old
4	TQ (Tu Qi)	10‐20‐2009	Female	JM (Jian Mao)	1–11 months old
5	HM (Hui Mao)	11‐10‐2009	Female	DY (Du Yan)	1–10 months old
6	DD (Dou Ding)	11‐12‐2009	Male	HB (Hua Bi)	1–10 months old
7	XY (Xiao Yao)	03‐23‐2010	Male	JG (Ji Guan)	1–6 months old

**TABLE A3:** Observation time (proportion) of play behaviors for each infant among different month of age.

Age	XC (Xiao Chou)	DM (Duan Mian)	XB (Xiao Bao)	TQ (Tu Qi)	HM (Hui Mao)	DD (Dou Ding)	XY (Xiao Yao)
1 month	30 (7.2%)	10 (16.3%)	40 (10.2%)	10 (16.7%)	70 (30.0%)	30 (10.9%)	20 (9.1%)
2 months	60 (22.0%)	90 (18.5%)	80 (22.4%)	110 (32.7%)	40 (21.3%)	80 (37.0%)	50 (23.5%)
3 months	180 (48.3%)	100 (26.4%)	60 (15.9%)	70 (35.4%)	70 (28.3%)	90 (34.9%)	230 (24.5%)
4 months	80 (52.1%)	120 (35.7%)	70 (29.7%)	40 (33.3%)	50 (44.1%)	60 (44.0%)	20 (40.2%)
5 months	87 (49.4%)	100 (30.5%)	40 (37.9%)	50 (56.3%)	90 (43.1%)	70 (48.9%)	22 (42.2%)
6 months	40 (48.4%)	60 (33.9%)	90 (27.9%)	50 (53.2%)	30 (47.7%)	20 (34.8%)	30 (31.8%)
7 months	90 (31.4%)	60 (46.0%)	50 (25.3%)	40 (30.6%)	80 (36.6%)	90 (42.3%)	—
8 months	30 (35.6%)	20 (9.3%)	50 (18.9%)	210 (25.0%)	30 (21.2%)	20 (28.2%)	—
9 months	70 (29.3%)	80 (21.0%)	20 (24.8%)	30 (22.0%)	10 (22.3%)	50 (31.0%)	—
10 months	20 (38.3%)	30 (25.8%)	40 (19.5%)	10 (37.2%)	10 (57.7%)	30 (40.7%)	—
11 months	30 (40.7%)	30 (19.6%)	20 (77.2%)	10 (40.3%)	—	—	—
12 months	30 (35.2%)	20 (55.6%)	—	—	—	—	—

*Note*: The unit of the observation time is second.

**TABLE A4:** Results of LMMs used to test the differences in the relative frequency and duration of play behaviors across different ages or sexes.

Models		BIC	LogLik	Deviance	Chisq
model1	data.null1: allfrequency ~ + (1 | ID)	407.13	−197.17	394.34	
	data.model1: allfrequency ~ age + (1 | ID)	382.40	−182.68	365.35	29.00
model2	data.null2: allduration ~ + (1 | ID)	369.31	−178.26	356.52	
	data.model2: allduration ~ age + (1 | ID)	320.93	−151.94	303.88	52.64
model3	data.null3: nonsocialfre ~ + (1 | ID)	362.19	−174.70	349.41	
	data.model3: nonsocialfre ~ age + (1 | ID)	340.60	−161.77	323.55	25.86
model4	data.null4: nonsocialdur ~ + (1 | ID)	403.94	−195.57	391.15	
	data.model4: nonsocialdur ~ age + (1 | ID)	389.94	−186.45	372.89	18.26
model5	data.null5: socialfre ~ + (1 | ID)	391.25	−189.23	378.46	
	data.model5: socialfre ~ age + (1 | ID)	274.75	−128.85	257.70	120.77
model6	data.null6: socialdur ~ + (1 | ID)	426.56	−206.89	413.77	
	data.model6: socialdur ~ age + (1 | ID)	344.31	−163.63	327.26	86.52
model7	data.null7: frequency57 ~ sex + (1 | ID)	386.64	−184.77	369.53	
	data.model7: frequency57 ~ age + sex + (1 | ID)	374.00	−176.31	352.62	2.92
model8	data.null8: frequency58 ~ sex + (1 | ID)	388.28	−185.59	371.17	
	data.model8: frequency58 ~ age + sex + (1 | ID)	390.85	−184.73	369.47	1.71
model9	data.null9: frequency59 ~ sex + (1 | ID)	417.05	−199.97	399.94	
	data.model9: frequency59 ~ age + sex + (1 | ID)	402.40	−190.51	381.01	18.93
model10	data.null10: frequency60 ~ sex + (1 | ID)	402.36	−192.63	385.26	
	data.model10: frequency60 ~ age + sex + (1 | ID)	391.47	−185.04	370.08	15.17
model11	data.null11: duration57 ~ sex + (1 | ID)	428.78	−205.84	411.68	
	data.model11: duration57 ~ age + sex + (1 | ID)	423.71	−201.16	402.32	1.35
model12	data.null12: duration58 ~ sex + (1 | ID)	455.62	−219.26	438.51	
	data.model12: duration58 ~ age + sex + (1 | ID)	458.80	−218.71	437.42	1.09
model13	data.null13: duration59 ~ sex + (1 | ID)	461.24	−222.07	444.14	
	data.model13: duration59 ~ age + sex + (1 | ID)	452.93	−215.77	431.54	12.59
model14	data.null14: duration60 ~ sex + (1 | ID)	453.96	−218.43	436.85	
	data.model14: duration60 ~ age + sex + (1 | ID)	452.56	−215.59	431.18	5.67

*Note*: ID means individual identity; BIC is Bayesian information criterion.
