# Corrigendum

**DOI:** 10.1002/ece3.9420

**Published:** 2022-10-11

In the recent article by Yang et al. (2022), the authors have confirmed that the Acknowledgment section should read as follows.
